# Contribution of the Mobilome to the Configuration of the Resistome of *Corynebacterium striatum*

**DOI:** 10.3390/ijms251910499

**Published:** 2024-09-29

**Authors:** Catherine Urrutia, Benjamin Leyton-Carcaman, Michel Abanto Marin

**Affiliations:** 1 Scientific and Technological Bioresource Nucleus (BIOREN), Universidad de La Frontera, Temuco 4811230, Chile; urrutiacatherine8@gmail.com (C.U.); leyton.bl@gmail.com (B.L.-C.); 2 Carrera de Biotecnología, Facultad de Ciencias Agropecuarias y Medioambiente, Universidad de La Frontera, Temuco 4811230, Chile; 3 Doctorado en Ciencias Mención Biología Celular y Molecular Aplicada, Universidad de La Frontera, Temuco 4811230, Chile

**Keywords:** *Corynebacterium striatum*, mobilome, mobile genetic elements, antimicrobial resistance, integrative and conjugative elements (ICEs)

## Abstract

*Corynebacterium striatum*, present in the microbiota of human skin and nasal mucosa, has recently emerged as a causative agent of hospital-acquired infections, notable for its resistance to multiple antimicrobials. Its mobilome comprises several mobile genetic elements, such as plasmids, transposons, insertion sequences and integrons, which contribute to the acquisition of antimicrobial resistance genes. This study analyzes the contribution of the *C. striatum* mobilome in the transfer and dissemination of resistance genes. In addition, integrative and conjugative elements (ICEs), essential in the dissemination of resistance genes between bacterial populations, whose role in *C. striatum* has not yet been studied, are examined. This study examined 365 *C. striatum* genomes obtained from the NCBI Pathogen Detection database. Phylogenetic and pangenome analyses were performed, the resistance profile of the bacterium was recognized, and mobile elements, including putative ICE, were detected. Bioinformatic analyses identified 20 antimicrobial resistance genes in this species, with the *Ermx* gene being the most predominant. Resistance genes were mainly associated with plasmid sequence regions and class 1 integrons. Although an ICE was detected, no resistance genes linked to this element were found. This study provided valuable information on the geographic spread and prevalence of outbreaks observed through phylogenetic and pangenome analyses, along with identifying antimicrobial resistance genes and mobile genetic elements that carry many of the resistance genes and may be the subject of future research and therapeutic approaches.

## 1. Importance

In recent years, clinical settings have increasingly faced outbreaks of multidrug-resistant bacteria, which primarily impact immunocompromised patients, leading to prolonged hospital stays, treatment complications, and even death. *C. striatum*, a persistent bacterium in clinical environments and human mucosa, is notable for harboring multiple antimicrobial resistance genes, complicating treatment and eradication. Much of the resistance gene spread occurs through mobile genetic elements (MGEs), contributing to the proliferation of these bacteria and difficult-to-control outbreaks. Our study aims to identify how antimicrobial resistance is disseminated through MGEs in *C. striatum*, enhancing our understanding of how the mobilome of multidrug-resistant bacteria contributes to the spread of resistance genes in clinical settings.

## 2. Introduction

The genus *Corynebacterium* belongs to the class Actinobacteria, order Actinomycetales, and family Corynebacteriaceae. These microorganisms are common, especially in soil and water, and some of them are naturally present on human skin and mucous membranes as commensal microbiota [1]. In the last decade, *C. striatum* has been frequently isolated and cultured from various surfaces and medical equipment in hospital settings [2]. *C. striatum* strains can form biofilms, which contributes significantly to their pathogenicity by facilitating colonization and infection [3]. Biofilm formation acts as a virulence mechanism that may allow some corynebacterial pathogens, including *C. striatum*, to adhere to living and artificial surfaces. Biofilm formation also appears to exert protective effects against antibiotics and the immune system [4].

However, the *Corynebacterium* species identified during laboratory procedures are still frequently considered contaminants of clinical specimens and/or underestimated by various healthcare professionals in many countries [5]. *C. striatum* is also an emerging pathogen in deep diabetic foot infections [6]. Moreover, there are risk factors associated with osteomyelitis in diabetic foot infections. These include prolonged prior antibiotic therapy, ischemic diabetic foot, and atrial fibrillation, all of which increase the likelihood of developing this type of infection [7].

Since 2000, *C. striatum* has been associated with nosocomial infections, which are frequently observed in immunocompromised patients hospitalized for long periods in intensive care units (ICUs) due to invasive medical devices such as intravenous catheters [5]. *C. striatum* is relatively resistant to antimicrobial therapy. While early reports indicated that *C. striatum* isolates were frequently susceptible to many antimicrobial drugs, including beta-lactams, tetracycline, and fluoroquinolones, more recent studies have shown an increase in multidrug resistance [8]. The mechanisms involved in antimicrobial resistance are becoming increasingly well understood. Resistance can be a natural property of a microorganism (intrinsic) or extrinsic by acquiring plasmids and transposons-related elements [5]. Horizontal gene transfer (HGT) remains a crucial driving force in bacterial evolution, especially in the spread of antimicrobial resistance (AMR). HGT mechanisms are mainly mediated by mobile genetic elements (MGEs), collectively known as the mobilome. Recently, the richness of the resistome in *C. striatum* has been evidenced, primarily associated with MGEs [9]. MGEs include integrative and conjugative elements (ICEs), plasmids, insertion sequences, transposons, prophages, integrons, and genomic islands [10]. 

Despite the identification of MGEs in *C. striatum*, to date it has not been explained with certainty which of these elements is primarily responsible for carrying genes associated with antimicrobial resistance, as not all antimicrobial resistance genes have been associated with MGEs in *C. striatum*. We hypothesized that antimicrobial resistance in *C. striatum* are encoded by MGEs, including integrative and conjugative elements (ICEs). Therefore, the main objective of this study was to identify the major MGEs present in the *C. striatum* mobilome that contribute to the transfer and dissemination of antimicrobial resistance genes.

## 3. Results

### 3.1. Resistance Profile in Corynebacterium striatum

**Figure 1:**
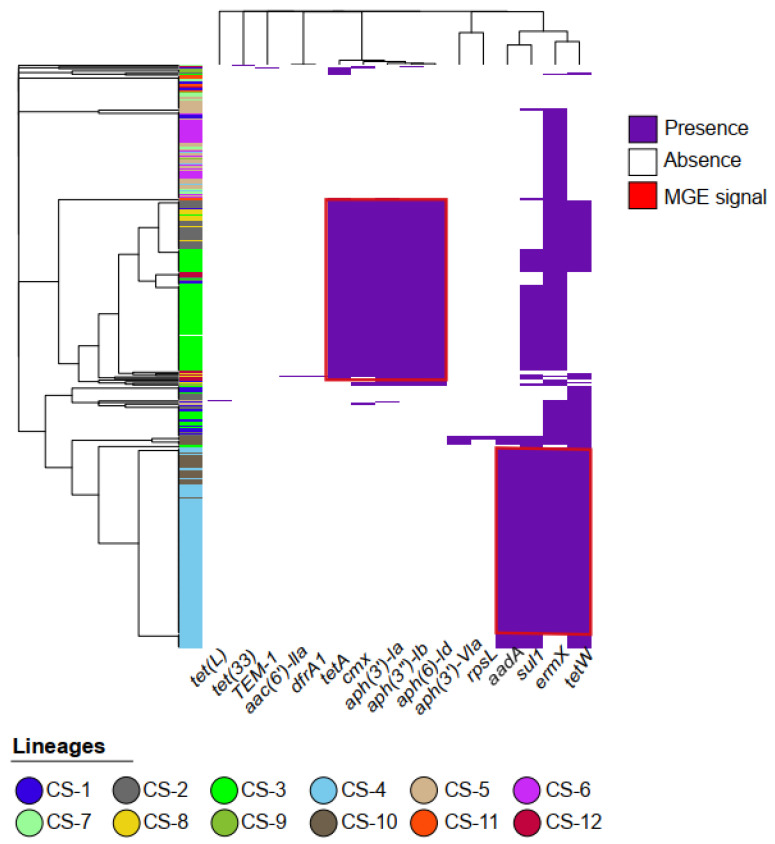
Genomic landscape of antibiotic resistance in *C. striatum*. The heatmap represents a binary matrix of presence (purple) and absence (white) of 20 antimicrobial resistance genes in 365 *C. striatum* genomes. Binary distance was used to calculate the similarity between resistance profiles, and the complete hierarchical clustering algorithm (complete linkage) was used to group genomes and genes. The dendrogram on the left shows the clustering of strains based on the similarity of their resistance profiles, while the upper dendrogram indicates the similarity in gene co-occurrence. The red boxes indicate possible MGEs, while the different lineages (please see the Figure 2) are represented in the dendrogram on the left.

Antimicrobial resistance (AMR) gene prediction using the sraX program v1.5 identified 20 AMR genes in the genomes analyzed (Table S1). The *ErmX* gene, associated with resistance to streptogramin, macrolides, and lincosamide, showed the highest prevalence, present in 314 of the 365 *C. striatum* genomes analyzed. Other common genes included a 16S rRNA mutation (hydromycin B resistance, n = 307), *tet(W)* (tetracycline resistance, n = 216), and *sul1* (sulfonamide resistance, n = 206). Importantly, while most of the AMR genes identified appear to be horizontally acquired, resistance conferred by mutations in the *16S*, *23S* (macrolide resistance), and *rpsL* (aminoglycoside resistance) genes results from changes in their respective rRNAs, representing a distinct intrinsic resistance mechanism. Subsequent analysis of AMR gene distribution and co-occurrence patterns (Figure 1) revealed two main clusters of consistently co-occurring genes. The first cluster includes *aadA*, *aac(6′)-IIa* (both associated with aminoglycoside resistance), *sul1*, and *tet(W)*, co-occurring in approximately 130 genomes. The second cluster includes *aph(6)-Id*, *aph(3′)-Ib*, *aph(3′)-Ia* (all conferring aminoglycoside resistance), *cmx* (chloramphenicol resistance), and *tetA* (tetracycline and penam resistance), co-occurring in about 100 genomes.

**Figure 2:**
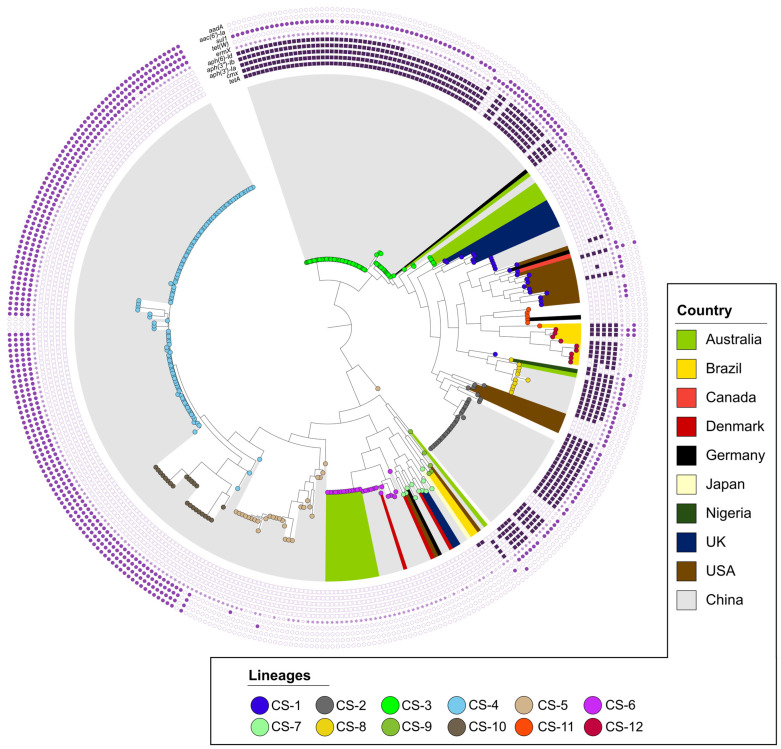
Population diversity and resistance in *C. striatum*. Bacterial populations were determined using RhierBAPS and are represented by colored circles in each tree tip. The most abundant antibiotic resistance genes are shown in purple and have been grouped into two categories according to their relationship or hypothetical origin: (1) circles for AMR genes related to pJA144188 and (2) squares for those related to pTP10. The tree was constructed using a GTR model with 1000 bootstrap replicates. Rooting (midpoint) and visualization were performed using the online tool iTol (https://itol.embl.de).

Phylogenetic and population structure analyses further revealed that *C. striatum* has 12 lineages (Figure 2), with CS-3 and CS-4 being the most important in terms of AMR gene storage. The CS-3 lineage, present in 83 genomes, shows resistance genes such as *tetA*, *cmx*, *aph(3′)-Ia*, *aph(3″)-Ib*, and *aph(6)-Id*. Meanwhile, the CS-4 lineage is present in 110 genomes and possesses the resistance genes *aadA*, *sul1*, *ErmX*, and *tet(W)*. These two lineages are the most abundant in *C. striatum* genomes and harbor the highest number of AMR genes, which may be because they are carried by mobile genetic elements (MGEs). 

### 3.2. Differential Resistance in Populations of C. striatum

Comparative genomic analysis of *C. striatum* strains revealed a complex population structure and considerable genome diversity. The phylogenetic study showed that the genomes share ~44% of core genes, indicating significant genomic variability. Pangenome analysis identified 1409 highly conserved core genes, 312 flexible core genes, and 1734 accessory genes, totaling 12,072 gene families. This genomic structure, combining a highly conserved gene set with a significant proportion of variable genes, reflects the ability of *C. striatum* to maintain essential functions while adapting to different ecological niches and selective pressures.

The branching structure of the tree (Figure 2) is evidence of genetic diversity within *C. striatum*. Multiple lineages (CS-1 to CS-12) were identified, some showing close genetic relatedness, while others diverged significantly. In addition, it was observed that lineages can spread geographically between different countries, such as the CS-1 lineage, found in China (5 genomes), the United States (12 genomes), the United Kingdom (7 genomes), Canada (1 genome), and Germany (1 genome), or CS-2, present in the United States (5 genomes) and China (25 genomes). There are also local or endemic lineages, such as CS-12 in Brazil, and lineages CS-2, CS-3, CS-4, and CS-5 in regions of China. In addition, some lineages, such as CS-2, CS-3, CS-4, and CS-6, are clonal and endemic. These findings indicate that *C. striatum* has a “mixed population structure”, exhibiting simultaneously cosmopolitan, endemic, and potentially epidemic characteristics. 

The phylogenetic tree illustrates this heterogeneous geographical distribution, with a predominance of isolates from China, particularly Beijing, followed by other cities such as Tangshan, Taiyuan, and Guangzhou (Figure S1). Potential epidemiological outbreak events, evidenced by clades with similar branch lengths and close temporality (2016–2018), were observed in several Chinese cities [11]. In addition, Figure S2 shows the differential gene content, highlighting those genomes in extreme clades, mainly from Beijing and Tangshan, share distinctive gene content profiles. Other cities that presented cases of *C. striatum* correspond to the city of Rio de Janeiro between the years 2009 to 2011; the city of Boston, where all its genomes are from the year 2016; and Queensland, all from the year 2019 [12]. Most of the genomes were isolated from clinical samples, predominantly sputum samples [13]. 

These results demonstrate that *C. striatum* has a mixed population structure. The heterogeneous geographical distribution and the observed genetic variability are a consequence of nosocomial transmission, human-mediated dispersal, and adaptation to specific ecological niches. The diversity in gene content between lineages indicates local adaptations that probably influence the pathogenicity and antimicrobial resistance of *C. striatum*. This genomic plasticity explains its epidemiological heterogeneity, allowing it to persist and spread in diverse clinical settings globally.

To assess the phylogenetic distribution of resistance profiles, we projected the identified AMR gene clusters onto the phylogenomic tree of *C. striatum* (Figure 2). This projection revealed a clear association between *C. striatum* populations and specific antimicrobial resistance patterns. This non-random distribution of AMR genes suggests a significant influence of population structure on the acquisition and maintenance of specific resistance profiles. In addition, two main clusters of consistently co-occurring AMR genes were identified, possibly associated with MGEs related to plasmids pJA144188 and pTP10, as indicated in the following section.

### 3.3. Probable Sources of Resistance in C. striatum

In order to determine the possible source and spread of antimicrobial resistance in *C. striatum* through mobile genetic elements, a comprehensive analysis of these elements, including bacteriophages and integrative and conjugative elements (ICEs), was performed. The identification of prophages by VIBRANT identified 105 prophage regions distributed in the analyzed genomes (Figure S3). Subsequent searching identified two putative ICEs. The first, ~43 kb, was located within a previously identified 150 kb prophage region, containing three integrases (Figure S4). The second putative ICE, ~237 kb, was characterized by the presence of *att* sites, a type IV secretion system, a relaxase and an integrase (Figures S4 and S5). Sequence similarity analysis revealed that these putative ICEs are integrated in different regions of the *C. striatum* genome, suggesting independent acquisition or insertion events (Figure S6). Functional characterization of the first ICE by HHpred did not reveal genes directly associated with antimicrobial resistance. Of the 62 proteins analyzed, a large number of them had unknown functions. The remaining proteins identified included transposases, recombinases, peptidases, and DNA-binding proteins, among others (Table S2). On the other hand, we also found no resistance genes in the 105 prophage regions identified. Although these mobile elements did not show a direct association with previously identified AMR genes, their presence and diversity in *C. striatum* underline the genomic plasticity in this species.

To elucidate the genomic organization and possible horizontal transfer of the identified AMR genes, a comprehensive analysis of the genomic context and the presence of mobile elements was conducted. Initial screening using UGENE revealed AMR gene clusters in specific regions. Subsequent sequence similarity analysis using BLAST against the NCBI database identified homology with plasmid sequences, which prompted a systematic investigation of plasmids associated with *C. striatum*.

A search of the PLSDB plasmid database yielded four plasmids that had been previously identified in *C. striatum.* The subsequent analysis with UGENE of genomes that shared specific resistance profiles (as delineated in Figure 1) revealed the presence of fragmented integration of these plasmids into bacterial genomes. Determination of the frequency of these plasmids in the population studied by using the LS-BSR tool v1.4 revealed that pCs-Na-2 (NZ_CP021254.1) was present in two genomes, pCs-Na-1 (CP021253.1) in 72 genomes, pTP10 (NC_004939.1) in 89 genomes, and the “*unnamed*” plasmid (CP069515.1) in 36 genomes. Notably, a fifth plasmid, pJA144188 (NC_014167.1), originally associated with *Corynebacterium resistens*, was identified in 232 of the 365 analyzed *C. striatum* genomes, constituting the most frequent plasmid element in the set of *C. striatum* genomes studied.

Gene content analysis revealed the presence of AMR genes in these plasmids. Specifically, pJA144188 carries six resistance genes (*ErmX*, *tet(W)*, *cmx*, *sul1*, *aadA*, and *aac(6′)-Ia)*; pTP10 contains five (*ErmX*, *tetA*, *cmx*, *aph(6)-Id*, and *aph(3′)-Ia)*; and the “*unnamed*” plasmid harbors one (*ErmX*). In total, 9 of the 20 AMR genes identified in *C. striatum* (*ErmX*, *tet(W)*, *cmx*, *sul1*, *aadA*, *aac(6′)-Ia*, *tetA*, *aph(6)-Id*, and *aph(3′)-Ia*) are present in plasmids. It is noteworthy that the two clonal clades of Chinese origin in the phylogenetic tree (Figure 2) consistently exhibited the presence of plasmid pJA144188. Furthermore, it was frequently observed that genomes lacking plasmid sequences specific to *C. striatum* contained elements of plasmid pJA144188 from *C. resistens*. It is noteworthy that some genomes showed evidence of the content of up to three different plasmid sequences, indicating a complex dynamic of acquisition and retention of mobile genetic elements. The high frequency of plasmid pJA144188, which originated from *C. resistens*, in *C. striatum* genomes studied here suggests interspecific transfer of resistance elements, thereby underscoring the potential for AMR gene dissemination between species of the genus *Corynebacterium*.

**Figure 3:**
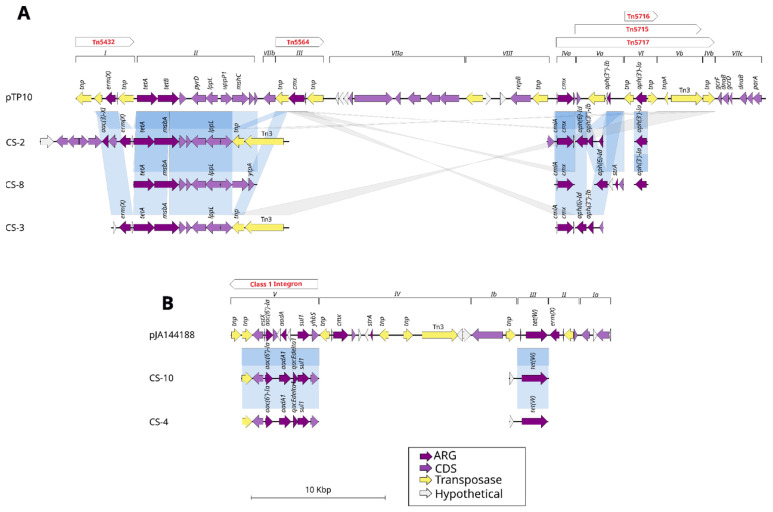
Comparison of plasmid sequences with *C. striatum* lineages. (**A**) Plasmid pTP10 is distinguished by modules in dark purple, which represent AMR genes. The regions where transposons are located in the plasmid are represented with red letters above the modules. The genomes belonging to each lineage CS-2, CS-8, and CS-3 are aligned in blue horizontal lines, which demonstrate the regions and genes they share. (**B**) The plasmid pJA144188, comprising five modules, has been aligned with the lineages CS-10 and CS-4. Module V contains a class 1 integron that is shared with the lineages, in addition to the resistance gene *tet(W)* in module III.

A comparison of plasmids pTP10 from *C. striatum* and pJA144188 from *C. resistens* (Figure 3) with previously identified lineages revealed that certain genes present in modules I through III of pTP10 are aligned and share identity hits exceeding 95% with the lineages CS-2 (32 genomes), CS-8 (11 genomes), and CS-3 (83 genomes). Furthermore, they carry between one and two genes found in the transposons Tn5432 and Tn5564, as well as AMR resistance genes such as the *ErmX* and *tetA* genes, while the last regions of the pTP10 plasmid ranging from module IVa to IVb and encompassing the transposons Tn5716, Tn5715, and Tn5717 share AMR genes such as *cmx*, *aph(3″)-Ib*, and *aph(3′)-Ia*. Regions VIIA and VIII are the only two regions of the plasmid that do not share genes with the analyzed lineages. However, there is a type 3 transposon (Tn3) from the pTP10 plasmid that is shared with lineages CS-2 and CS-3.

In the case of plasmid pJA144188, there are two modules (V and III) that share alignments with lineages CS-10 (23 genomes) and CS-4 (110 genomes). Module V, which also contains the class 1 integron, shares a transposon and the AMR genes *aac(6′)-Ia*, *aadA* and *sul1*, while in module III of the plasmid, the gene *tet(W)* is shared. 

### 3.4. Characterization of C. striatum Plasmids

**Figure 4:**
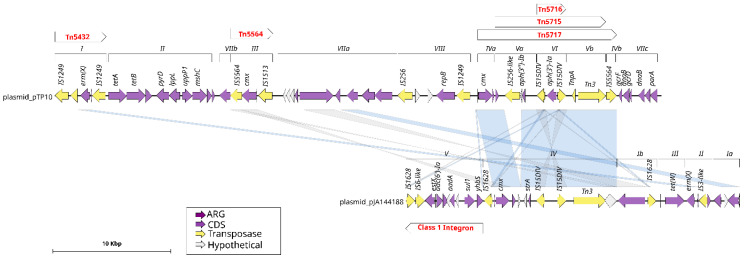
Similarities of plasmids pTP10 from *C. striatum* and pJA144188 from *C. resistens*. The purple arrows indicate the coding sequence (CDS) of the pTP10 plasmid, while the numbers above them represent the plasmid modules. Transposons are shown with red letters. The pTP10 plasmid is aligned with the pJA144188 plasmid, as indicated by the blue lines, which represent regions that share similarities between these plasmids.

To enhance comprehension of these mobile genetic elements, a comparative analysis of the structure and gene content of the plasmids identified in the genomes of *C. striatum* was conducted (Figure 4). The analysis demonstrated that a significant proportion of the plasmid modules are comprised of transposases. Furthermore, conserved regions were identified among the “*unnamed*” plasmid, pJA144188, pTP10, and pCs-Na-1. These regions included genes encoding replication proteins present in all four plasmids, as well as common transposases and resolvases. This conservation suggests a shared evolutionary origin or horizontal transfer events among these elements.

In particular, plasmids pJA144188 and pTP10 exhibited a high degree of similarity, with a total of eight genes in common. Plasmid pJA144188 and the “*unnamed*” plasmid shared a total of five genes. It is noteworthy that plasmid pCS-Na-2 was distinguished from the other plasmids analyzed by the absence of any homologous regions, which suggests that it may have a distinct evolutionary origin or have been acquired independently.

The plasmid pJA144188, originally characterized in *C. resistens* by Schröder in 2012 [14], was subjected to comprehensive analysis, resulting in the identification of its five functional modules and the six resistance genes it harbors. A comparison of the structural and functional characteristics of the pJA144188 plasmid with those of the pTP10 plasmid from *C. striatum* (Figure 4) revealed that module IV of pJA144188 shares several regions with pTP10, including the *cmx* resistance gene. Additionally, a substantial number of genes present in modules ranging from the regions of module IVa to IVb of pTP10 that are aligned with genes from module IV in plasmid pJA144188 also carry three transposons belonging to pTP10: Tn5716, Tn5715, and Tn517. This finding suggests that recombination or gene transfer events may have occurred between these plasmids. Additionally, it was identified that module V of pJA144188 corresponds to a class 1 integron carrying the resistance genes *sul1*, *aadA*, and *aac*(*6′)-Ia*, which are known to play a role in the distribution and propagation of antimicrobial resistance [15].

## 4. Discussion

In this study, an analysis was performed of the contribution of the mobilome in the configuration of the resistome of *C. striatum*. Multiple analyses were conducted to achieve this objective. The initial analyses were meant to comprehensively understand the bacterium’s evolution by conducting a phylogenetic study. This study revealed that the majority of *C. striatum* genomes originated in China between 2016 and 2018, particularly in Beijing and Tangshan. These findings agree with the results presented by Kang [16], who conducted a study to identify clonal strains of *C. striatum* and identified a greater number of clonal groups (CGs) in these two cities, possibly due to their geographic proximity. Importantly, the straight-line distance between Beijing and Tangshan is only 155 km, suggesting a possible influence of geography on the distribution of *C. striatum* clonal groups [16]. This observation highlights the importance of considering geography as a relevant factor in the dispersion and spread of the bacterium [17]. Most of the *C. striatum* genomes we worked with came from clinical isolates from sputum samples, and this may be caused by nosocomial outbreaks of *C. striatum* that were mainly due to prolonged hospitalizations, repeated exposures to broad-spectrum antibiotics, and prolonged use of invasive medical devices [18].

According to previous reports that *C. striatum* is a colonizer of skin and mucous membranes [8], it is predominantly found in clinical samples of sputum and secretions, especially during the summer, and shows a tendency for rapid spread and evolution in different clinical departments [19].

In the pangenome analysis for the identification of differential gene content, the results identified 12,072 gene families in the 365 genomes of *C. striatum*; of these, 1409 belong to the core genomes, and 1734 belong to shell genes present in 15% to 95% of the genomes. This could be attributed to the moderate expansion of the shell genome set, which suggests the presence of an open pangenome in this species. This expansion is likely a result of the incorporation of genes from other species, which provide a successful survival strategy for the bacterial population to maintain competitiveness in their respective environments [11,20].

In the susceptibility analysis, to determine the antimicrobial resistance profiles through the sraX program, 20 resistance genes were detected in *C. striatum*, possibly due to intrinsic or extrinsic mechanisms. Intrinsic mechanisms are natural phenomena in all bacteria; they are generally obtained by regulating membrane permeability and non-specific efflux pumps. In contrast, extrinsic or generally acquired mechanisms are received by the horizontal transfer of mobile elements [9]. The antimicrobial resistance genes due to intrinsic mechanisms were genes *16s*, *23s*, and the *rpsL* gene that present mutations in the rRNA. Therefore, the remaining 18 resistance genes may be related to MGEs. The resistance genes that exhibited the highest number were the *ErmX*, *tet(W)*, and *sul1* genes. This result is consistent with the findings of Kang [16], which turned out to be similar to ours in the high percentage of these genes in *C. striatum* isolates, in addition to presenting resistance genes such as *aac(6′)-IIa*, *aac(6′)-Ib7*, *and aph(3′)-VIa* genes in lower percentages.

Studies have shown that *C. striatum* isolates are resistant to penicillin, meropenem, ceftriaxone, tetracycline, clindamycin, erythromycin, and ciprofloxacin [21]. This is consistent with our results because the antimicrobial resistance genes found are largely resistant to these antimicrobials, such as the *ErmX* gene, which is associated with streptogramin antibiotics, lincosamide antibiotics, and macrolide resistance; the *sul1* gene, which confers resistance to sulfonamide antibiotics; the *tet(W)* gene, which confers resistance to tetracycline; and the *aac(6′)-IIa*, *aac(6′)-Ib7*, and *aph(3′)-VIa* genes, which confer resistance to aminoglycosides [16].

With regard to the lineages identified in the genomes of *C. striatum*, two main lineages (CS-3 and CS-4) were found to be responsible for harboring the greatest number of AMR genes. This revealed that 193 genomes out of 365 present one of these two lineages, which possess multi-resistance to various classes of antibiotics. The aforementioned lineages (CS-3 and CS-4) are predominantly sourced from China. A study published in 2021 examined *C. striatum* from respiratory samples of hospitalized patients in three hospitals in China. Four lineages were identified that are believed to have emerged 10–20 years ago and subsequently disseminated throughout the country, transmitted via nosocomial infections and resulting in a national epidemic [22]. However, our results also indicate that the CS-3 lineage is not exclusive to Chinese genomes. In fact, it has also been identified in clinical samples from Australia dating back to 2019. However, the AMR genes they possess are limited to *ErmX* and *tet(W)*, indicating that this lineage has disseminated to other regions, rendering it a potential pandemic lineage with the capacity to disseminate between different countries. Furthermore, our findings indicate that this lineage is not confined to a single geographical region. The CS-1 lineage has been identified in China and the United Kingdom, while the CS-2 lineage has been observed in the United States and China. The majority of the identified lineages are present in China and are clonal in nature. Some lineages, such as CS-2 (32 genomes) and CS-8 (11 genomes), also present several AMR genes; however, their prevalence is less pronounced than that of the CS-3 and CS-4 lineages. In contrast, a lineage presenting multiple AMR genes, including *tetA*, *cmx*, *aph(3′)-Ia*, *aph(3″)-Ib*, *aph(6)-Id*, *ErmX*, and *sul1*, has been identified that does not originate from China. This lineage, CS-12, has been observed in nine genomes from Brazil. Therefore, we believe that *C. striatum* has a “mixed population structure”, highlighting the importance of seeking epidemiological surveillance tools for this worrying bacterium and to help prevent potential public health emergencies.

To evaluate the source of antimicrobial resistance in *C. striatum*, we focused on resistance genes that were clustered only in certain genomes and represented the highest percentage of antimicrobial resistance genes because they could be transported by MGEs since in *C. striatum*, the dissemination of antimicrobial resistance genes is associated with MGEs such as plasmids, transposons, and insertion sequences [9]. The detection of four regions of plasmid sequences specific to *C. striatum* was achieved; these are pCs-Na-1, pCs-Na-2, pTP10, and “*unnamed*”. Of these, plasmid pTP10 is the one that carries the highest number of antimicrobial resistance genes with five resistance genes and is present in 89 genomes. The presence of the multi-resistance plasmid pTP10 explains the resistance of this bacterium to macrolides, tetracyclines, and clindamycin [23]. A review by Leyton and Abanto [24] notes that only four plasmids have been recorded in *C. striatum* and are the same as those found in the A plasmid database. pTP10 plasmid is the best known: it was reported in *C. striatum* M82B from a 1983 strain. However, there are no studies related to the other three plasmids [24].

Although most genomes studied are fragmented into multiple contigs, we did not identify complete plasmids other than those few already reported. However, in our results, we identified a plasmid region that does not belong to *C. striatum*, is present in 232 of the 365 genomes analyzed in this study, and carries six antimicrobial resistance genes. This plasmid, designated pJA144188 and belonging to the pathogenic bacterium *C. resistens*, was isolated from a positive blood culture from a patient with acute myelocytic leukemia [14]. This plasmid also contains a region associated with a class 1 integron and contains the *sul1*, *aadA*, and *aac(6′)-la* resistance genes found in one-third of the 365 analyzed *C. striatum* genomes. Commonly, integrons are characteristic of Gram-negative bacteria; only a few integrons have been reported from Gram-positive bacteria [14]. Integrons are genetic elements containing a site-specific recombination system capable of integrating, expressing, and exchanging specific DNA elements, called genetic cassettes, which are often exposed to mechanisms that allow them to spread horizontally through bacterial populations [25]. In a study by Hendor [26], a pangenome analysis of *C. striatum*, including isolates from the skin microbiome and MDR infections, was performed to gain insight into genetic factors contributing to pathogenicity and multidrug resistance in this species. They managed to find a class 1 integron carrying the *sul1*, *qacE*, *aadA*, and *aac(6)-lb7* genes, with the *sul1* and *aadA* genes being the same as those carried by the class 1 integron found in plasmid pJA144188. Therefore, plasmid sequence regions and class 1 integrons would be among the major contributors responsible for the propagation of antimicrobial resistance genes in *C. striatum*.

The plasmids found in the *C. striatum* genomes were characterized with the GenoFig program v1.1, obtaining the most relevant result that the plasmid pJA144188 of *C. resistens* and pTP10 of *C. striatum* share several modules, including the *cmx* resistance gene and some transposases. In the study conducted by Schröder [14], where the plasmid pJA144188 was found and characterized, the authors assigned module I of the plasmid as the one that possessed similarity to the multidrug resistance plasmid pTP10 of the human opportunistic pathogen *C. striatum*. However, in our study, it was found that the module that shares the most similarity between the two plasmids is module IV, which they associated with the Tn 5717c transposon, which is highly similar to the Tn 5717a transposon of the *C. urealyticum* DSM 7109 chromosome [14], suggesting that this transposon has been transported between species of the genus *Corynebacterium*.

In the search for ICEs in *C. striatum*, two filters were performed: the first was for bacteriophages, which present similar characteristics to those of phages since they integrate and replicate with the host chromosome [27]. As a result, 105 bacteriophages were obtained, which were then filtered a second time to search for any possible ICEs, finding only one putative ICE, which possessed three integrases, two at the ends and one in the middle of the element. Integrases determine the insertion site (or sites) of the ICE and, frequently, excision from the host chromosome [28]. The putative ICE was characterized, and each of its proteins was searched for its functionality. Resolvases, integrases, kinases, and genes related to adaptive capacity were found. However, this putative ICE was not found to be related to antimicrobial resistance in *C. striatum*.

Hence, we believe that the putative ICE found may have other beneficial functions for the bacterium because ICEs are not only related to antimicrobial resistance but may also contain phenotypes that contribute to pathogenicity or metabolic capabilities [29]. In addition, many ICEs can mobilize other genetic elements, typically plasmids, that do not encode their own transfer machinery [30]. ICEs have also been discovered in bacterial genomes that protect the host from bacteriophage predation [31].

This in silico study signifies a noteworthy advance that opens fresh perspectives for future research in the field of antimicrobial resistance, focusing on emerging pathogens. Using bioinformatics tools, we identified and understood the overall picture of a nosocomial pathogen such as *C. striatum* and its mobilome, which is responsible for the maintenance and dispersal of antimicrobial resistance genes among bacterial populations. This study has improved our understanding of how the *C. striatum* mobilome influences the configuration of the bacterial resistome in clinical settings. This information is relevant as the bacterium has spread from China to other countries, including Brazil, where nosocomial outbreaks of *C. striatum* carrying resistance genes have been reported. This situation underscores the importance of identifying and studying the presence of *C. striatum* in our country and region. The next step would be to carry out this work in an in vivo setting, which would be of great relevance. Obtaining genomes from the local population would make it possible to identify the presence of antimicrobial resistance in bacteria and understand what types of elements are facilitating their transfer. This preventive approach would help avoid possible nosocomial outbreaks in the future and the transfer of resistance to other pathogenic bacteria. In addition, it would provide information to determine which antibiotics would be effective in case the bacterium possesses certain resistance genes.

## 5. Materials and Methods

### 5.1. Genome Recovery

We retrieved 365 *C. striatum* genomes including assembled genomes and raw sequences from the NCBI Pathogen Detection database (https://www.ncbi.nlm.nih.gov/pathogens) (accessed on 5 January 2023). Unicycler [32] was used to assemble the raw sequences, after quality filtering with fastp [33] and a visual check with FastQC v0.11.9 [34]. Of the 365 genomes, 358 were from clinical samples, predominantly from sputum and blood.

### 5.2. Bioinformatics Analyses

A phylogenetic analysis based on SNPs of core genes was performed using Harvest suite tools v1.1.2 [35]. For core genome alignment, the FDAARGOS_1115 strain (Genbank: GCF_016728105.1) was used as a reference. Then, from the parsnp results, a tree was constructed using IQTRE v1.6.12 [36] with a GTR model and 1000 bootstrap replicates. Allelic populations in *C. striatum* were calculated with Rhierbaps v1.0.1 [37,38].

To study the differential gene content, we proceeded to study the pangenome of *C. striatum*. For this, the genomes were annotated using Prokka v1.14.5 [39], and then the genetic repertoire was analyzed with Roary v3.11.2 [40], which was run by default with the parameters of minimum percentage identity for BLASTP of -i 95 and a percentage of isolates for core genes: -cd 99.

In order to identify possible ICEs, a search for prophages was carried out, since they have mechanical, structural, and genetic similarities between them, and their ancestral evolutionary relationships are discussed [41]. VIBRANT v1.2.1 [42] was used to search for prophages in *C. striatum* genomes. To reduce redundancy of prophage sequences, CD-HIT v4.8.1 [43] was used, with an 80% identity threshold. Representative prophage sequences were submitted to VIPtree v4.0 [44] for phylogenetic analyses. Finally, these prophage sequences were analyzed with ICEfinder v2.6.32 [45] to characterize possible ICEs. The results were then explored using Proksee v1.3.1 [46] and HHpred v2.08 [47].

### 5.3. Data Analysis and Visualization

The R package pheatmap version 1.0.12 [48] with parameters such as hierarchically grouped row and column clustering was used to identify patterns of ARG content similarity between genomes. Phylogenetic results were explored in iTOL v6.9.1 [49]. Genomic exploration was carried out with Proksee v.1.3.1 [46] and Unipro UGENE v47.0 [50]. To analyze and compare the plasmids of *C. striatum*, GenoFig v1.1 [51] was used. Then, to compare genomes of the different lineages of *C. striatum* with the plasmids found, the genomes were annotated using the web version of Bakta v1.9.1 [52] and then compared with GenoFig v1.1.

### 5.4. Genomic Analysis of Resistome and Mobile Elements

Antimicrobial resistance genes were predicted using the sraX program v1.5 [53] with parameters such as percent identity at 95% and with the other default parameters. Heatmap analyses segregated resistance gene clusters in analyzed *C. striatum* genomes (see Section 3). To associate these gene clusters with mobile genetic elements, we explored the genomic context of these genes with Unipro UGENE v47.0 [50] via blastn [54]. ARG-associated mobile elements were then searched across all genomes with LS-BSR v1.4 [55] with a threshold of 80% identity. In turn, we retrieved *C. striatum*-associated plasmids contained in the PLSDB database (https://ccb-microbe.cs.uni-saarland.de/plsdb, accessed on 10 September 2023) [56] and searched all genomes with LS-BSR with a threshold of 80% identity. Finally, the identified plasmids were compared to Genofig v1.1 with 0.8 identity.

## 6. Conclusions

In this study, we analyzed the contribution of the mobilome in the configuration of the resistome of *C. striatum* by bioinformatics analysis. We identified 20 antimicrobial resistance genes with extrinsic resistance genes predominating in the bacterium. These are transported mainly by plasmid sequence regions, such as plasmid pTP10, but mostly by the plasmid of the pathogenic bacterium *C. resistens* pJA144188 and its class 1 integron carrying the *sul1*, *aadA*, and *aac(6′)-la* resistance genes. ICEs and prophages are not elements responsible for carrying antimicrobial resistance genes in *C. striatum*; however, to confirm these findings, additional analyses are needed to identify what benefits they provide to this bacterium.

## Data Availability

The genomic data and associated information used in this research were retrieved from the NCBI Pathogen Detection Database (https://www.ncbi.nlm.nih.gov/pathogens) and can be accessed through the accession numbers described in the supplementary table.

## Supplementary Materials

The following supporting information can be downloaded at: https://www.mdpi.com/article/10.3390/ijms251910499/s1.
