# Supplementary material for: Contribution of the Mobilome to the Configuration of the Resistome of Corynebacterium striatum
Source: Int J Mol Sci. 2024 Sep 29;25(19):10499. doi: 10.3390/ijms251910499 (PMC11477358; doi:10.3390/ijms251910499)
Supplement: Supplementary file 1 [file ijms-25-10499-s001.zip › Supplementary figures.pdf]

Supplementary Figures

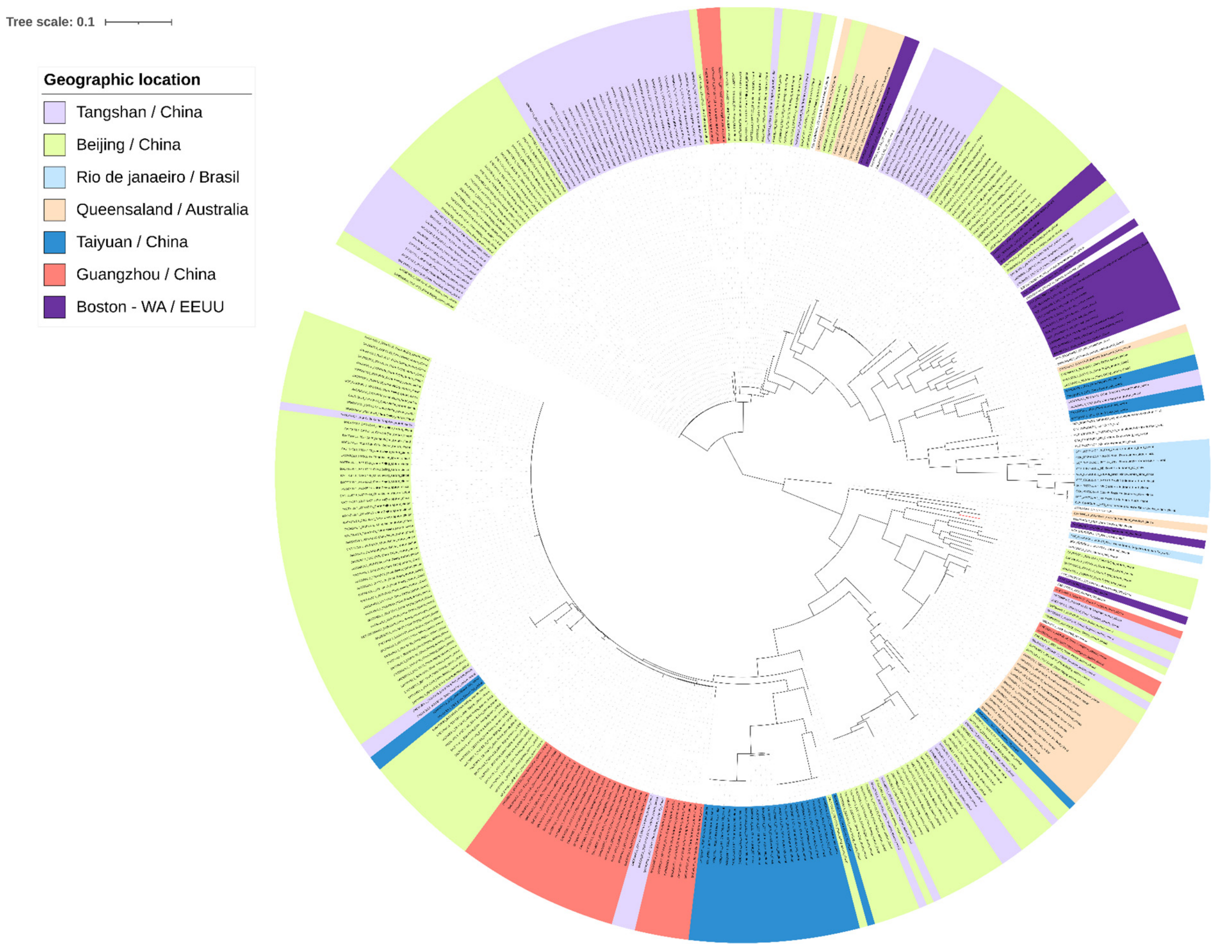

Figure S1: Phylogenetic tree and geographic locations of *C. striatum* genomes. Phylogenetic tree with the 365 genomes of *C. striatum*. The colours represent the geographical region of each genome. The most representative geographical areas belong to localities in China, specifically Tangshan (purple) and Beijing (yellow).

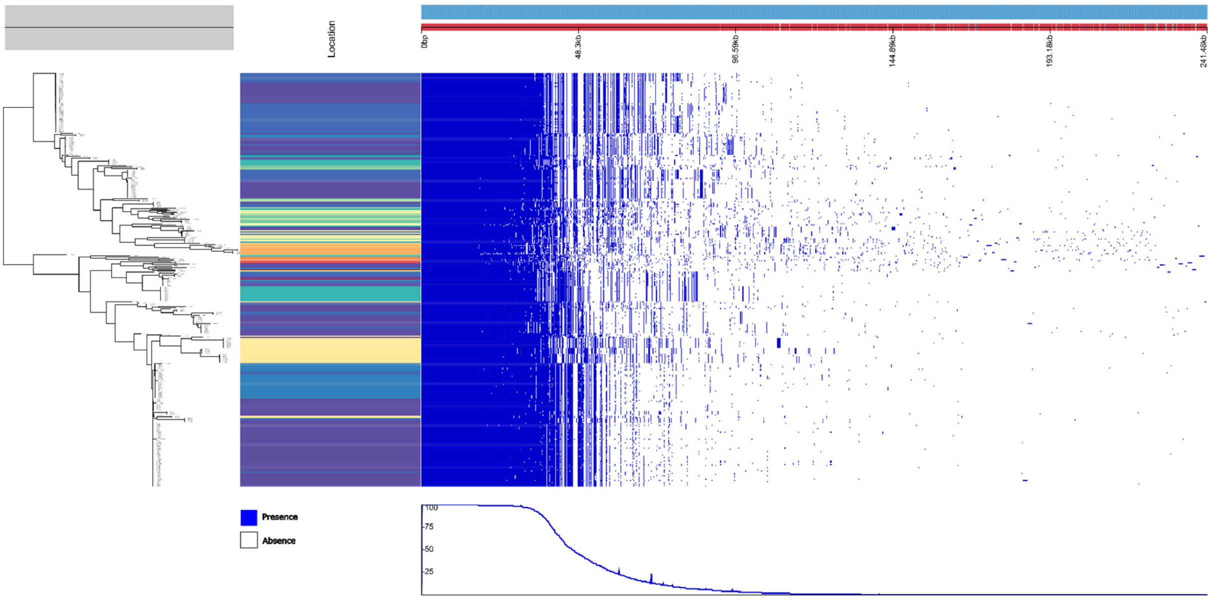

Figure S2: Pangenome graph and differential gene content. The pangenome and phylogenetic tree graph shows the differential gene content in *C. striatum*, with blue showing the presence of genes and white showing their absence. In addition, the metadata of the geographical location is added where the colours ranging from purple to light blue represent the regions of China that predominate in the graph, the specific cities are Beijing in purple, Tangshan in blue and Guangzhou in light blue, these regions being found in greater proportion between the first clades and the last of them.

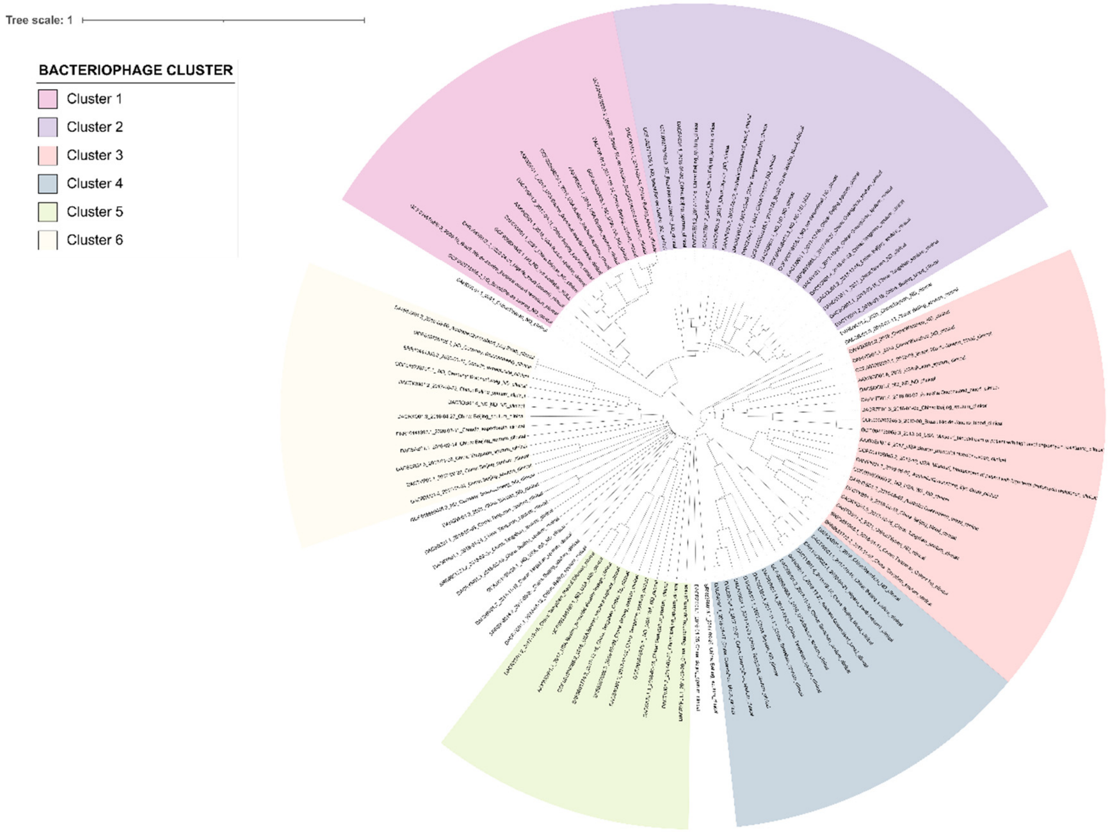

Figure S3: Phylogenetic tree of representative bacteriophages from *C. striatum* genomes. Evolutionary analysis of bacteriophages from phage prediction by Vibrant was performed on 365 *C. striatum* genomes. A total of 105 bacteriophages were identified and then divided into six distinct colored clades.

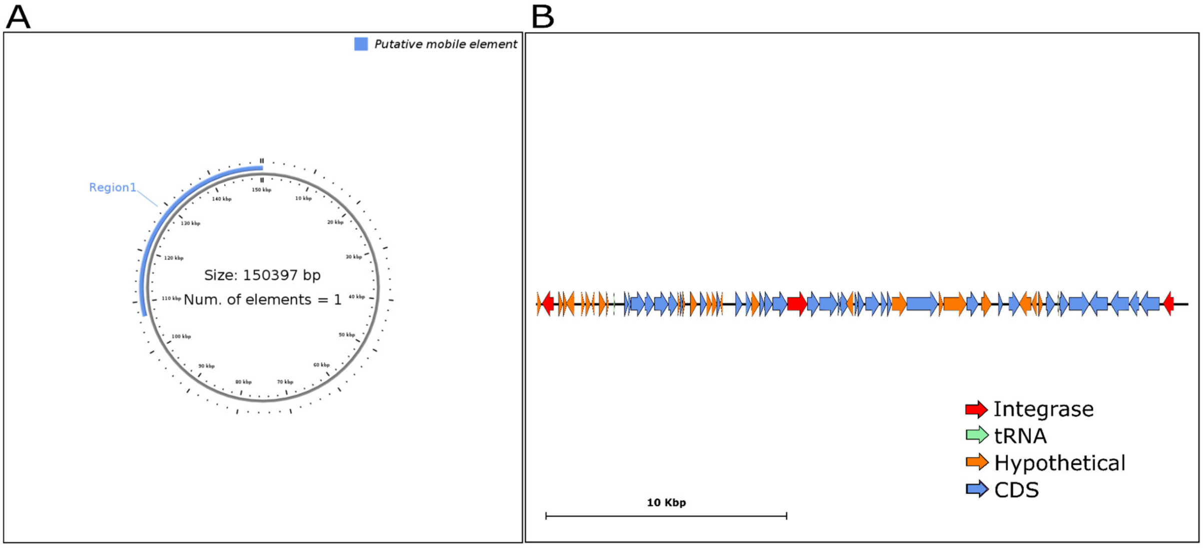

Figure S4: Putative ICE 1. Putative ICE identified by ICEfinder tool, region 1 corresponds to the ICE within a 150 kb bacteriophage (A). The element with a genome of 43 kb has 3 integrases inside it (B).

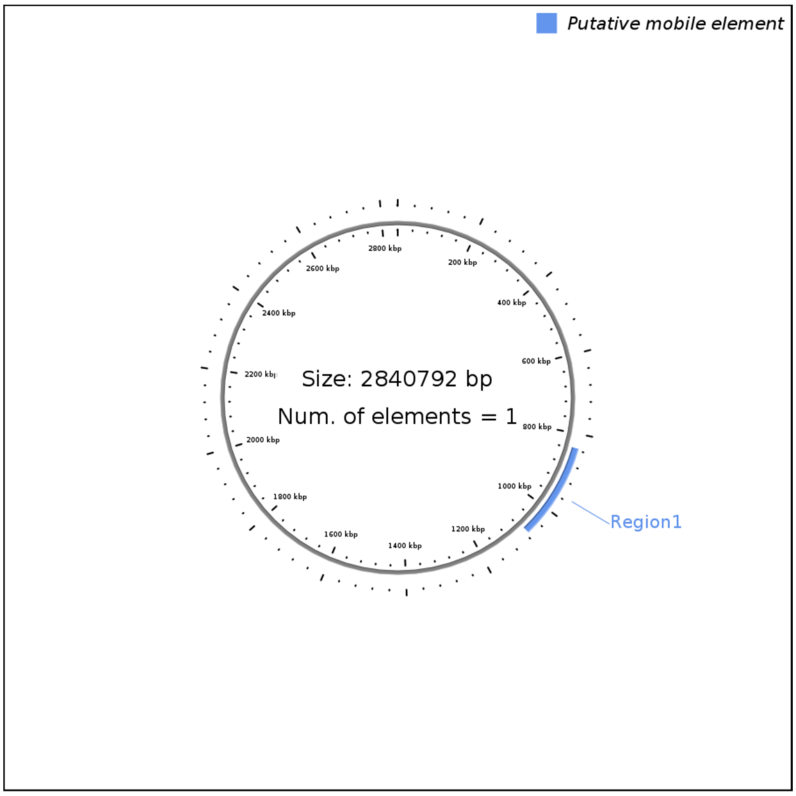

Figure S5: Second putative ICE in *C. striatum* genome. ICE identified by ICEfinder tool, region 1 corresponds to the 237 kb ICE within a *C.striatum* genome of 2.8 Mb in size, this genome is the same as the one in the bacteriophage where the first putative ICE was found.

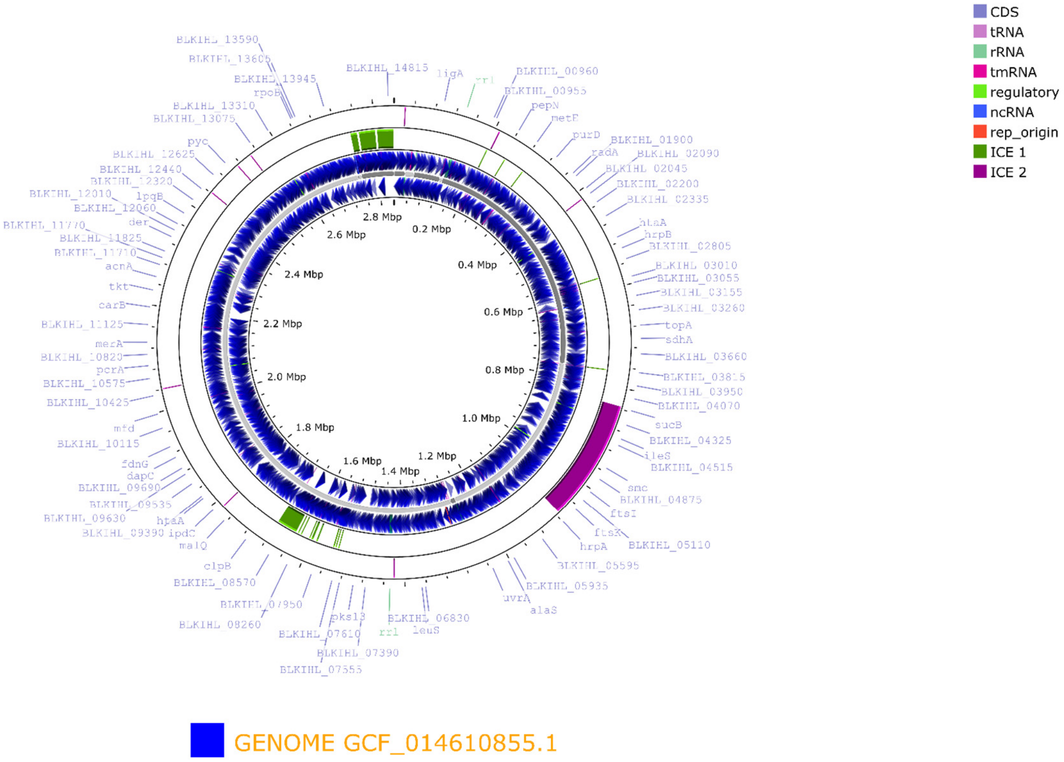

Figure S6: Putative ICEs in *C. striatum* genome. Two putative ICEs found by ICEfinder in the same genome of *C. striatum* GCF_014610855 In green is the first ICE identified with a size of 43kb which is inserted in the genome in two different regions and in purple is the second ICE identified with a size of 237 kb.
